# Establishment and optimization of a liquid bead array for the simultaneous detection of ten insect-borne pathogens

**DOI:** 10.1186/s13071-018-2996-0

**Published:** 2018-07-31

**Authors:** Hui-yu Wang, Shao-qiang Wu, Li Jiang, Rong-hai Xiao, Ting Li, Lin Mei, Ji-zhou Lv, Jia-jia Liu, Xiang-mei Lin, Xue-qing Han

**Affiliations:** Chinese Academy of Inspection and Quarantine, Beijing, 100176 People’s Republic of China

**Keywords:** Insect-borne pathogens, Liquid array, Optimization, Multiplex

## Abstract

**Background:**

Insect-borne diseases could induce severe symptoms in human and clinical signs in animals, such as febrility, erythra, arthralgia and hemorrhagic fever, and cause significant economic losses and pose public health threat all over the world. The significant advantages of Luminex xMAP technology are high-throughput, high parallel and automation. This study aimed to establish a liquid bead array based on Luminex xMAP technology that was able to simultaneously detect multiple insect-borne pathogens.

**Methods:**

Specific probes and primers to detect the nucleic acid of 10 insect-borne pathogens were designed. Probes were coupled with fluorescent carboxylated microspheres. The parameters of the system were optimized, including ratio of forward/reverse primers (1:2), hybridization temperature (50 °C) and duration (30 min) and quantity of PCR product (2 μl). The sensitivity and specificity of the system were also evaluated. Moreover mixed nucleic acid of 10 insect-borne pathogens, including Bluetongue virus, Epizootic hemorrhagic disease virus of deer, *Coxiella burnetii*, African swine fever virus, West Nile fever virus, *Borrelia burgdorferi*, vesicular stomatitis virus, Rift Valley fever virus, Ebola virus and Schmalenberg’s disease virus, and 3000 clinical samples were tested for practicability.

**Results:**

The optimized detection system showed high sensitivity, specificity and reproducibility. Each probe showed specific fluorescence signal intensity without any cross-hybridization for the other insect-borne pathogens tested, which included dengue virus, tick-borne encephalitis virus, Japanese encephalitis virus, Xinjiang hemorrhagic fever virus, spotted fever group rickettsiae, ehrlichiae and chikungunya virus. The limit of detection was 10 copies of target gene. Insect-borne pathogens were successfully detected among the 3000 clinical samples, and the results were consistent with those obtained using gold-standard assays or commercial nucleic acid detection kits.

**Conclusions:**

This optimized liquid array detection system was high-throughput and highly specific and sensitive in screening of the insect-borne pathogens. It was promising in detection of these pathogens for molecular epidemiological studies.

**Electronic supplementary material:**

The online version of this article (10.1186/s13071-018-2996-0) contains supplementary material, which is available to authorized users.

## Background

Insect-borne diseases are infectious diseases spread among animals and human through hemophagia by blood-feeding arthropods, such as mosquitoes, ticks and midges, which are widespread in the environment [1]. These infections lead to significant human and animal morbidity and mortality worldwide [1], which cause a huge economic loss. In recent years, epidemic outbreaks of insect-borne infectious diseases in countries neighboring and trading with China have posed a threat to public health [2], especially in the border areas of China. Many insect-borne diseases in the border areas of China have drawn public attention, such as West Nile virus (WNV) infection in Xinjiang [3], Tahyna virus infection in the Qinghai-Tibet Plateau [4], and mosquito-borne arbovirus (e.g. Japanese encephalitis virus and Sindbis virus) infection in Yunnan [5]. It is remains difficult to develop effective vaccines against such viruses [6]. Furthermore, the clinical symptoms of many infections by mosquito-borne arboviruses do not indicate a specific pathogen, and some infections are even asymptomatic [7]. Therefore, accurate and timely diagnosis of these infections is a great challenge of utmost importance.

To date, multiple molecular detection methods have been established to detect insect-borne pathogens, including reverse transcriptase-polymerase chain reaction (PCR) [8], real-time PCR [9], a liquid bead array [10] and a microwell membrane array [11]. Recent studies have established modified PCR or array methods for the detection of insect-borne pathogens [11–13]. However, these methods are only able to detect one type or a few types of viruses, which greatly restricts their application. Luminex xMAP technology is a multiplexed high-throughput detection system that uses fluorescent carboxylated microspheres. The Luminex array offers up to 100 independent channels and use microspheres embedded with various ratios of two fluorescent dyes. A mixed suspension of microspheres is mixed with the sample to bind analytes, which are then labeled with a fluorescent reporter and analyzed using a specialized flow cytometer [14, 15].

In this study, we established a method that was able to simultaneously detect multiple insect-borne pathogens rapidly and effectively, including bluetongue virus (BTV), epizootic hemorrhagic disease virus of deer (EHDV), Q-fever pathogen *Coxiella burnetii* (CB), African swine fever virus (ASFV), West Nile fever virus (WNV), *Borrelia burgdorferi* (BB), vesicular stomatitis virus (VSV), Rift Valley fever virus (RVFV), Ebola virus (EBV) and Schmallenberg virus (SBV). This optimized liquid array detection system may contribute to the rapid and effective detection of multiple insect-borne diseases at border ports in China.

## Methods

### Target genes and fragments

Armored RNA technique was utilized in this study. The exogenous RNA fragments packaged by the virus-like particles (VLPs) expression vector pTMSCA2C were used as positive control of BTV, EHDV, WNV, VSV, RVFV, EBV and SBV.

The segment of the *NS1* gene of BTV was most closely related to the virus isolate CAR1982/04 (GenBank: KP 822059.1) from the Pirbright Institute, with 97% identity. The segment of the *NS3* gene of EHDV was most closely related to the virus isolate EHD1/USA2006/LA/CC332-06 (GenBank: KU140939.1), with 99% identity. The segment of *E* gene of WNV was most closely related to the West Nile virus isolate 876 (GenBank: KY229074.1), with 99% identity. The segment of *N* gene of VSV was most closely related to the virus isolate Colorado/21793/2014 (GenBank: KP 202364.1) from the National Veterinary Services Laboratories of USA, with 96% identity. The segment of *S* gene of RVFV was most closely related to the Rift Valley fever virus strain ZH-501 (GenBank: DQ380149), with 98% identity. The segment of *NP* gene of EBV was most closely related to the Sudan Ebola virus isolate EboSud-682 2012 (GenBank: KC545392.1), with 99% identity. The segment of *N* gene of SBV was most closely related to the virus isolate BH80/11-4 (GenBank: KC545392.1) from FLI of Germany, with 99% identity.

Target gene segments of ASFV, CB and BB were synthesized by Beijing Genomics Institute (BGI), Beijing, China. Then, 3 plasmids, containing above 3 target segments, were constructed as a positive control for further works.

The segment of *P72* gene of ASFV was most closely related to the African swine fever virus E75 (GenBank: FN557520), with 99% identity. The segment of *IS1111a* gene of CB was most closely related to the *Coxiella burnetii* NMII RSA439 (GenBank: M80806), with 98% identity. The segment of *OspA* gene of BB was most closely related to the *Borrelia burgdorferi* SZ (GenBank: AY502600), with 99% identity.

Other insect-borne pathogens, including dengue virus (DENV), tick-borne encephalitis virus (TBEV), Japanese encephalitis virus (JEV), Xinjiang hemorrhagic fever virus (XHFV), spotted fever group rickettsiae (SFGR), and ehrlichiae and chikungunya virus (CHIKV) used for specificity test, were cultured and conserved by Chinese Academy of Inspection and Quarantine.

### Oligonucleotide design

Based on the specific sequences of each pathogen in the GenBank database, primers and probes (capture probes and reverse-complement (RC) probes) were designed using different software packages, such as DNAMAN, MEGA, BioEdit, DNAStar and Primer Premier 5.0 (Table 1). Before designing the primers and probes, we collected available complete nucleotide sequences of target gene of each pathogen as many as possible. Multiple sequence alignments of each pathogen were analyzed and performed using different software, such as DNAMAN, MEGA. Specific primers and probes for each pathogen were selected from the conserved regions of each target gene. The National Center for Biotechnology Information (NCBI) BLAST network server was used for sequences homology analyzing. The specificity of the designed primers and probes was evaluated using Primer-BLAST on the NCBI website. All of the primer and probe sequences were found to exhibit high specificity. Alignment analysis in ClustalW showed that the level of homology between the probe sequences was less than 40%. Each probe was then checked for potential hybridization to any other, non-target sequences, amongst those amplified during multiplex PCR, by aligning its sequence to the sequences of all of the amplified products. The synthesis of primers and probes was conducted by Sangon Biotech Co., Shanghai, China.

**Table 1:** Primer and probe sequences for the 10 kinds of pathogen

Pathogen	Primer or probe sequence	Amplified fragment length (bp)	GenBank ID
ASFV	Forward primer: 5'-AGTTATGGGAAACCCGACCC-3'	254	FN557520
	Reverse primer: Biotin-5'-CCCTGAATCGGAGCATCCT-3'		
	Capture probe: NH_2_-C_12_-5'-AGCCTTATGTTCCAGTAGGGTTTG-3'		
	RC-probe: Biotin-5'-CAAACCCTACTGGAACATAAGGCT-3'		
BTV	Forward primer: 5'-GCAGCATTTTGAGAGAGCGA-3'	101	KP 822059.1
	Reverse primer: Biotin-5'-CCCGATCATACATTGCTTCCT-3'		
	Capture probe: NH_2_-C_12_-5'-GGAGGAGCCGGCGAAAGCATA-3'		
	RC-probe: Biotin-5'-TATGCTTTCGCCGGCTCCTCC-3'		
BB	Forward primer: 5'-AAGAGCAGACGGAACCAGAC-3'	345	AY502600
	Reverse primer: Biotin-5'-GTGCCATTTGAGTCGTATTG-3'		
	Capture probe: NH_2_-C_12_-5'-AATTCAGGCACTTCAACTTTAACAA-3'		
	RC-probe: Biotin-5'-TTGTTAAAGTTGAAGTGCCTGAATT-3'		
EHDV	Forward primer: 5'-TGATTATGATGTTCATGGCGAA-3'	189	KU140939.1
	Reverse primer: Biotin-5'-ACCTTGGAGCTTCACTCTATCT-3'		
	Capture probe: NH_2_-C_12_-5'-TAGATGGATTTGACATACCGCCGGA-3'		
	RC-probe: Biotin-5'-TCCGGCGGTATGTCAAATCCATCTA-3'		
VSV	Forward primer: 5'-TGGACGGGCTTGAAAATCAGTGCAAA-3'	199	KP 202364.1
	Reverse primer: Biotin-5'-TTGAATCTGGAAACAATAGTTCCGTATCT-3'		
	Capture probe: NH_2_-C_12_-5'-ATGTTCTTCCACATGTTCAAAAA-3'		
	RC-probe: Biotin-5'-TTTTTGAACATGTGGAAGAACAT-3'		
WNV	Forward primer: 5'-GAGCCACTCAGGCAGGGAGATTCAG-3'	496	KY229074.1
	Reverse primer: Biotin-5'-AACACCACAGTGCCGTGACCT-3'		
	Capture probe: NH_2_-C_12_-5'-TGAAGGGAACAACCTATGGCGTCTG-3'		
	RC-probe: Biotin-5'-CAGACGCCATAGGTTGTTCCCTTCA-3'		
CB	Forward primer: 5'-TGAGATTCGGGGGTTGTTGC-3'	376	M80806
	Reverse primer: Biotin-5'-ACACCTCCTTATTCCCACTCG-3'		
	Capture probe: NH_2_-C_12_-5'-TTTAACGGCGCTCTCGGTTTATGCG-3'		
	RC-probe: Biotin-5'-CGCATAAACCGAGAGCGCCGTTAAA-3'		
RVFV	Forward primer: 5'-ATGATGACATTAGAAGGGA-3'	298	DQ380149
	Reverse primer: Biotin-5'-ATGCTGGGAAGTGATGAG-3'		
	Capture probe: NH_2_-C_12_-5'-ATGCTGTAGTTCCAAACTCAGCCCT-3'		
	RC-probe: Biotin-5'-AGGGCTGAGTTTGGAACTACAGCAT-3'		
EBV	Forward primer: 5'-CGCTGGCTGGTGTTAATGTAGGG-3'	249	KC545392.1
	Reverse primer: Biotin-5'-ATGCAGTCGTGATGGCTTCGG-3'		
	Capture probe: NH_2_-C_12_-5'-CAGCAAACTAACGCAATGGTAACCT-3'		
	RC-probe: Biotin-5'-AGGTTACCATTGCGTTAGTTTGCTG-3'		
SBV	Forward primer: 5'-GAAGCTAGTGCTCAGATTGTCATGC-3'	130	KC545392.1
	Reverse primer: Biotin-5'-GTGGATAGAAGTCAAAAGCATCAAGG-3'		
	Capture probe: NH_2_-C_12_-5'-AAGGGATGCACCTGGGCCGATGGTTA-3'		
	RC-probe: Biotin-5'-TAACCATCGGCCCAGGTGCATCCCTT-3'		

*Abbreviations*: *ASFV* African swine fever virus, *BTV* bluetongue virus, *BB Borrelia burgdorferi*, *EHDV* epizootic hemorrhagic disease virus, *VSV* vesicular stomatitis virus, *WNV* West Nile virus, *CB Coxiella burnetii*, *RVFV* Rift Valley fever virus, *EBV* Ebola virus, *SBV* Schmallenberg virus

In order to avoid interference by different primers and templates, we designed more than 3 pairs of primers for each pathogen. Primers were designed to meet the following conditions: (i) amplification segments were between 100–300 bp to insure the intensity of fluorescence signal; (ii) melting point is between 50–60°C; and (iii) there are no long dimers and complimentary stem to insure the compatibility in a multiplex-PCR system.

### Extraction and amplification of nucleic acid

Total RNA or DNA of pathogens was extracted and purified using the QIAamp Viral RNA Mini kit or the QIAamp DNA Blood Mini kit (Qiagen, Valencia, CA, USA) according to the manufacturer’s protocol. The quality and concentration of RNA and DNA were evaluated by the ratios of A260/A280 and A260/A230 using a NanoDrop2000 spectrophotometer (NanoDrop2000, UV-vis spectrophotometer, USA). Subsequently, eligible RNA was reverse-transcribed into cDNA using the PrimeScript RT-PCR Kit (TaKaRa, Dalian, China).

Multiplex asymmetric PCR was applied to DNA amplification. The PCR system was determined as follows: 23 μl buffer mix, 0.5 μl forward primers (10 μmol/ml each), 0.5 μl reverse primers (10 μmol/ml each), 5 μl nucleic acid templates, added with double-distilled H_2_O up to 50 μl. The thermal cycle conditions were as follows: 1 cycle at 50 °C for 30 min and 95 °C for 15 min, followed by 35 cycles at 95 °C for 40 s, 54 °C for 40 s and 72 °C for 1 min, and a final extension at 72 °C for 10 min. Each reaction was repeated in triplicate. PCR products were confirmed by 2% agarose gel electrophoresis.

### Coupling of probes and micro-spheres

According to the manufacturer’s protocol, capture probes modified with an amino-C12 linker at the 5'-end were coupled with 10 types of Luminex MicroPlex fluorescent carboxylated micro-spheres (“beads”) (Luminex, Austin, TX, USA). Briefly, for each coupling of probe and bead set, 200 μl of 1.25 × 10^7^ beads/ml beads were re-suspended in 0.1 mol/l MES buffer (morpho-line ethane sulfonic acid, 50 μl, pH 4.5, Sigma-Aldrich, St. Louis, MO, USA), with the probe (3 μl of 0.1 nmol/μl stock to give 0.3 nM final concentration), and treated twice with 1-ethyl-3-(3-dimethylaminopropyl)-carbodiimide hydrochloride (EDC, 2.5 μl of a 10 mg/ml solution, Thermo Fisher Scientific/Pierce, Rockford, IL, USA) at room temperature away from light for 30 min. The mixture was then rinsed in Tween 20 (1 ml of a 0.02% aqueous solution), followed by sodium dodecylsulfate solution (SDS, 1 ml of a 0.1% solution), and re-suspended in Tris-EDTA buffer (pH 8.0) to the final volume of 100 μl. The final concentration of the bead suspension was calculated using a haemocytometer.

### Detection of coupling efficiency

According to the manufacturer’s (Bio-Rad, Hercules, CA, USA) protocol, the prepared suspension of beads coupled with capture probes was diluted to a final concentration of ≥ 100 beads/μl using 1× tetramethylammonium chloride (TMAC) hybridization solution. Then, 33 μl of diluted bead suspension was mixed with 15 μl Tris-EDTA buffer (pH 8.0) and 1 μl of different concentrations (1000, 500, 100, 50, 10 and 1 fmol/μl) of RC-probes modified with biotin at the 5'-end. Then, the mixtures were subjiected to PCR amplification (95 °C for 5 min, 54 °C for 30 min, maintenance at 4 °C) and the PCR products were transferred into a 96-well ELISA plate. The beads were re-suspended in 100 μl of streptavidin-R-phycoerythrin (SAPE) diluted in 1× TMAC hybridization solution (3:1,000, the final concentration: 3 μg/ml), and incubated in a metal bath at 58 °C for 10 min. Then, the reaction systems were analyzed using a Bio-Plex multiplexed liquid array detection system (Bio-Rad, Hercules, CA, USA). Each reaction was repeated in triplicate. The coupling efficiency of beads and probes was evaluated based on the median fluorescent intensity (MFI).

### Hybridization assay

The PCR products of pathogens were hybridized with probe-coupled beads using the liquid array. Briefly, the working solution was then prepared by mixing 1 μl of the 10 types of probe-coupled bead suspensions and 330 μl of 1× TMAC hybridization solution. Then, 1 μl of the positive PCR product of each virus was mixed with 33 μl of working solution and 15 μl of Tris-EDTA buffer (pH 8.0) in a tube. The PCR product in the blank control tube was replaced by 1 μl of double-distilled H_2_O, the negative control tube by 1 μl of negative PCR product, and the positive control tube by 1 μl of RC-probes (0.01 μmol/l). Each reaction was repeated in triplicate. The PCR amplification and subsequent steps were the same as those described for coupling efficiency detection. The MFI was computed to assess the results according to the manufacturer’s protocol. The Luminex qualitative ratio result (LQRR) was equal to the MFI of the sample (MFIS) divided by the MFI of the blank control (MFIB), that is LQRR = MFIS/MFIB. If LQRR ≥ 3, the result was positive; if LQRR < 2, the result was negative; and if 2 ≤ LQRR < 3, the result was inconclusive and needed to be reanalyzed. The procedures used for the suspension array method are outlined in Additional file 1: Figure S1.

### Optimization of hybridization conditions

To optimize the ratio of forward/reverse primers in the asymmetric PCR, five ratios of forward/reverse primers (1:1, 1:2, 1:3, 1:4 and 1:5) were tested. All other conditions remained constant.

To optimize the quantity of PCR product for hybridization efficiency, six concentrations of PCR product quantity (0.5, 1, 2, 4, 6 and 8 μl) were tested. The optimized ratio of forward/reverse primers, determined above, was used in the PCR, and all other conditions remained constant.

To optimize the melting temperature (Tm) for hybridization efficiency, six different Tm values (50, 52, 54, 56, 58 and 60 °C) were tested. The optimized ratio of forward/reverse primers and the optimized quantity of PCR product, determined above, were used in the PCR, and all conditions remained constant.

To optimize the hybridization duration, six different durations (15, 20, 25, 30, 35 and 40 min) were tested in the hybridization assay. The optimized ratio of forward/reverse primers, PCR product quantity and Tm, as determined above, were used in the PCR, and all other conditions remained constant.

### Establishment of the calibration curve

After the detection method had been optimized, 10 standard curves were established for the MFI values obtained from the experiments. A 10-fold dilution of pathogens was added to the system to create correlative mathematical curves with MFI values.

### Analysis of the sensitivity, specificity and reproducibility of the detection system

Each of the 10 pathogens and other insect-borne pathogens including DENV, TBEV, JEV, XHFV, SFGR and CHIKV were detected to determine the specificity of the liquid array detection system.

To determine the sensitivity of the liquid array detection system, nucleic acid of the pathogens was extracted using a Qiagen kit (Qiagen, Chatsworth, CA, USA). After detection of both the concentration and purity of the nucleic acid, the nucleic acid concentration of each pathogens was adjusted to the same magnitude (10^10^ copy number), then serially diluted with different folds (10–10^10^-fold ). The serially diluted nucleic acids were detected using liquid array method.

DNA or RNA templates for the 10 pathogens were detected in triplicate to evaluate the reproducibility of the liquid array detection system. The coefficient of variation (CV) was calculated as CV = (standard deviation (SD)/mean) × 100%.

### Detection of clinical samples

A total of 3000 samples of mosquitoes, ticks and midges provided by the Entry-Exit Inspection and Quarantine departments at five border areas in China (Table 2) were used for blind validation of the detection efficiency of the established liquid array detection system. All of the samples were then reanalyzed using gold-standard assays or existing nucleic acid detection kits (Table 3).

**Table 2:** Information on the nucleic acid samples used for clinical sample detection

Sampling area	Sample category	Sample number	Month
Inner Mongolia Autonomous Region	Mosquitoes	350	June-July
	Ticks	200	April-June
	Midges	230	June-July
Xinjiang Uighur Autonomous Region	Ticks	270	April-June
	Mosquitoes	300	June-July
Yunnan Province	Mosquitoes	320	May-August
	Midges	200	June-September
Heilongjiang Province	Mosquitoes	290	June-August
	Ticks	100	April-June
	Midges	220	June-July
Guangdong Province	Mosquitoes	300	March-June
	Midges	220	May-September
Total		3000

**Table 3:** Analysis of positive samples using the optimized liquid array detection system and corresponding reference methods or nucleic acid detection kits

Pathogens	Results of the two methods		Description of reference method or nucleic acid detection kit
	Optimized liquid array detection system	Reference methods or nucleic acid detection kit	
ASFV	0	0	PCR and agarose gel electrophoresis (primers recommended by OIE)
BTV	53	53	BTV nucleic acid detection kit^a^
BB	16	16	*Borrelia burgdorferi* nucleic acid detection kit^b^
EHDV	0	0	Virus isolation
VSV	0	0	VSV RT-PCR detection kit^c^
WNV	0	0	WNFV real-time PCR detection kit^d^
CB	17	17	Isolation of pathogen
RVFV	0	0	RVFV nucleic acid detection kit^e^
EBV	0	0	EBV nucleic acid detection kit^f^
SBV	0	0	Virotype SBV real-time RT-PCR kit(FLI-B585)^g^

^a^BTV nucleic acid detection kits and the real-time fluorescence quantitative PCR method were purchased from Beijing Senkang Biotechnology Development Co., Ltd., Beijing, China

^b^*Borrelia burgdorferi* nucleic acid detection kits and the PCR-fluorescent probe method were purchased from Guangzhou Vipotion Biotechnology Co. Ltd., Guangzhou, China

^c^VSV RT-PCR detection kits were purchased from YUABIO Biotechnology Co., Beijing, China

^d^WNFV real-time PCR detection kits were purchased from Beijing Biolab Technology Co. Ltd.

^e^RVFV nucleic acid detection kits and the real-time fluorescence quantitative PCR method were purchased from Beijing Senkang Biotechnology Development Co., Ltd., Beijing, China

^f^EBV nucleic acid detection kits and the PCR-fluorescent probe method were purchased from DA AN GENE Co. Ltd., Guangzhou, China

^g^Virotype SBV real-time RT-PCR kit (FLI-B585) was purchased from Qiagen, Valencia, CA, USA

## Results

### Coupling efficiency of beads and viral probes

The capture probes on the breads and the corresponding RC-probes of the 10 pathogens showed a high hybridization signal. With the concentration of RC-probes gradually decreasing, the MFI also gradually declined (Additional file 2: Figure S2), indicating that the coupling of beads and probes was successful.

### Results of optimizations

**Fig. 1:**
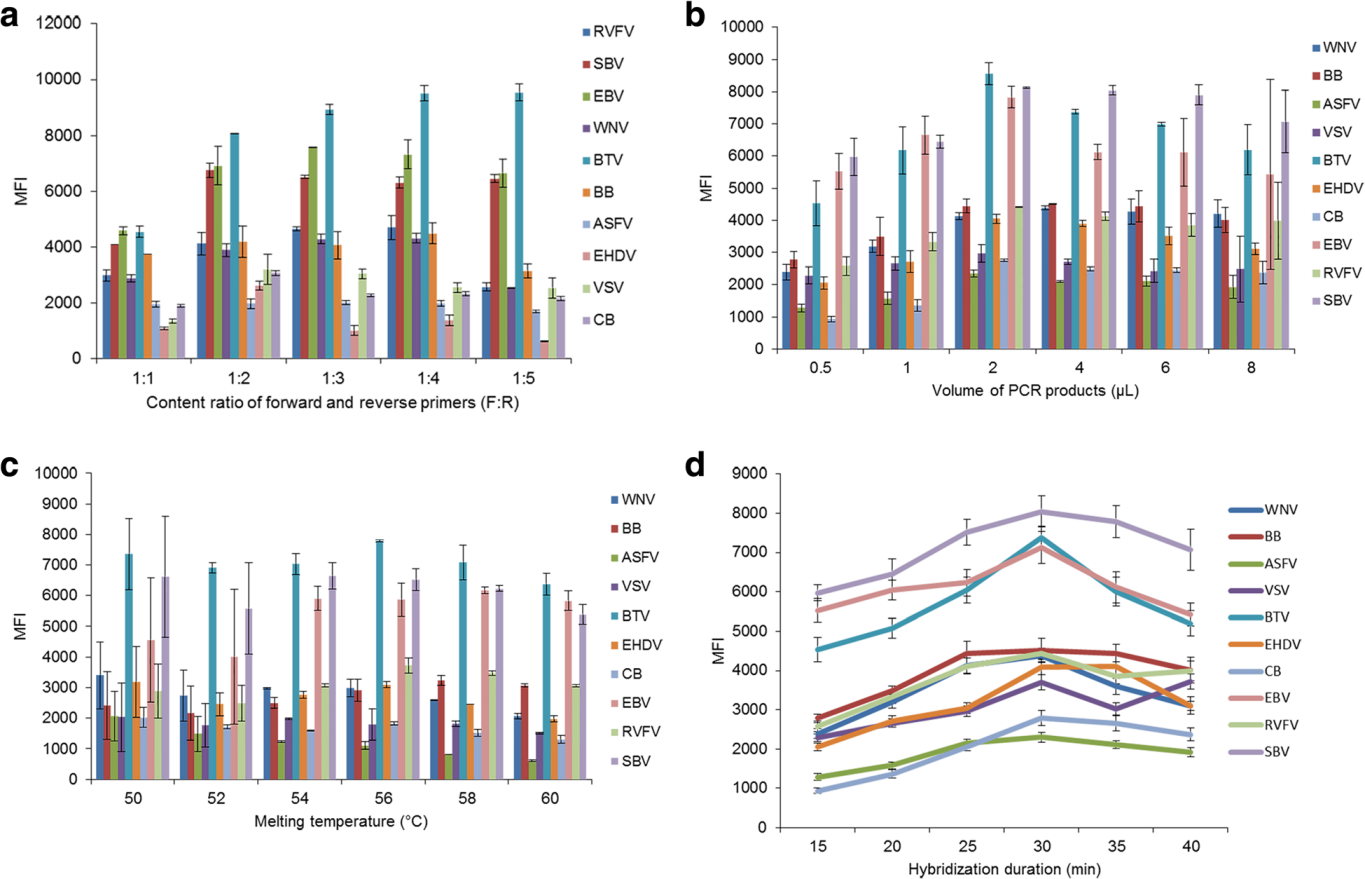
Median fluorescent intensity (MFI) for the 10 insect-borne pathogens with different ratios of forward/reverse primers, quantities of PCR product, melting temperatures for the hybridization and hybridization durations. **a** MFIs for different ratios of forward/reverse primers. **b** MFIs for different quantities of PCR product. **c** MFIs for different melting temperatures for the hybridization. **d** MFIs for different hybridization durations

Liquid array detection showed that the MFI for RVFV, SBV, EBV, WNV and BTV was relatively higher (mainly > 4000) when the ratio of forward/reverse primers was 1:2, 1:3 or 1:4. The MFI for EHDV, VSV and CB was the highest when the ratio of forward/reverse primers was 1:2, whereas the MFI for BB and ASFV was relatively stable regardless of the forward/reverse primer ratio (Fig. 1a). As a result, 1:2 was used as the optimized ratio of forward/reverse primers.

The MFI for BTV, EHDV, VSV, EBV and SBV PCR product was highest when the volume of PCR product was 2 μl, then gradually decreased with increasing volume of PCR product (> 2 μl). Similarly, the MFI for WNV, BB, ASFV, CB and RVFV PCR product was also highest when the volume of PCR product was 2 μl, but showed no obvious decrease with increasing volume of PCR product (> 2 μl) (Fig. 1b). Therefore, 2 μl of PCR product was selected as the optimized quantity for the asymmetric PCR.

The MFI for BTV PCR product was higher at 50 °C and 56 °C. The MFI for BB and EBV PCR product was highest at 58 °C, and for the other seven pathogens was highest at 50 °C (Fig. 1c). Therefore, 50 °C was selected as the optimized Tm for the hybridization.

The MFI for BB and EHDV PCR product was higher when the hybridization duration was 30 min or 35 min, whereas the MFI for the other eight pathogens was highest when the hybridization duration was 30 min (Fig. 1d). Therefore, 30 min was chosen as the optimal hybridization duration.

Above all, an optimized liquid array detection system was described as below. The asymmetric PCR system was set up as follows: 23 μl buffer mix, 0.5 μl forward primers, 1 μl reverse primers, 5 μl nucleic acid templates, and double-distilled H_2_O up to 50 μl. The thermal cycle conditions were as follows: 1 cycle at 50 °C for 30 min and 95 °C for 15 min, followed by 35 cycles at 95 °C for 40 s, 54 °C for 40 s and 72 °C for 1 min, with a final extension at 72 °C for 10 min. The hybridization system was set up as follows: 33 μl of diluted bead suspension (1 μl of probe-coupled bead suspension added into 330 μl 1× TMAC hybridization solution), 15 μl Tris-EDTA buffer (pH 8.0), and 2 μl PCR products. The hybridization reaction was performed as follows: denaturation at 95 °C for 5 min, and hybridization at 50 °C for 30 min. After hybridization, PCR products were transferred into a 96-well ELISA plate. Beads were re-suspended in 100 μl of SAPE diluted in 1× TMAC hybridization solution (3:1000), and incubated in a metal bath at 58 °C for 10 min. Then the reaction systems were analyzed using a liquid array detection system.

### Calibration curve

**Fig. 2:**
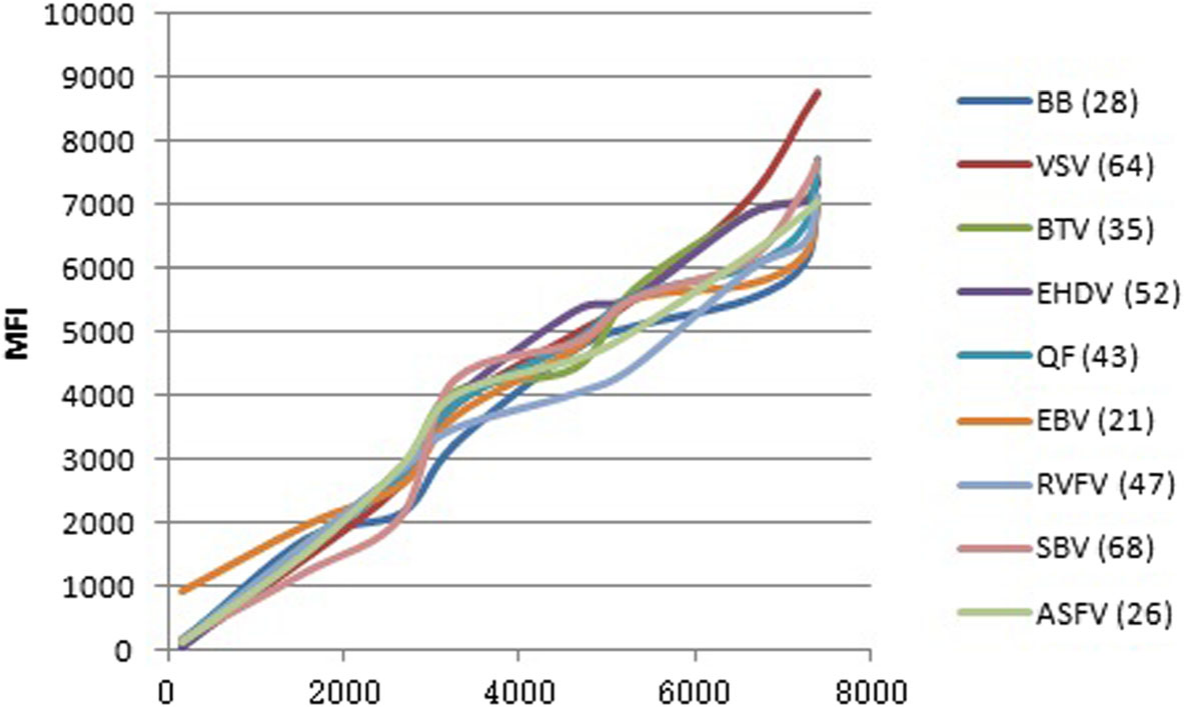
Calibration curves for each pathogen. A 10-fold dilution of pathogens was added to the system to create correlative mathematical curves with MFI values. All of the resulting correlation coefficients (*R*^2^) were greater than 0.95, indicating good relationships between the concentration of the target and fluorescence

After the detection method had been optimized, 10 standard curves were established for the MFI values obtained from the experiments. A 10-fold dilution of pathogens was added to the system to create correlative mathematical curves with MFI values (Fig. 2). All of the resulting correlation coefficients (*R*^2^) were greater than 0.95, indicating good relationships between the concentration of the target and fluorescence.

### Specificity, sensitivity and reproducibility of the liquid array detection system

**Fig. 3:**
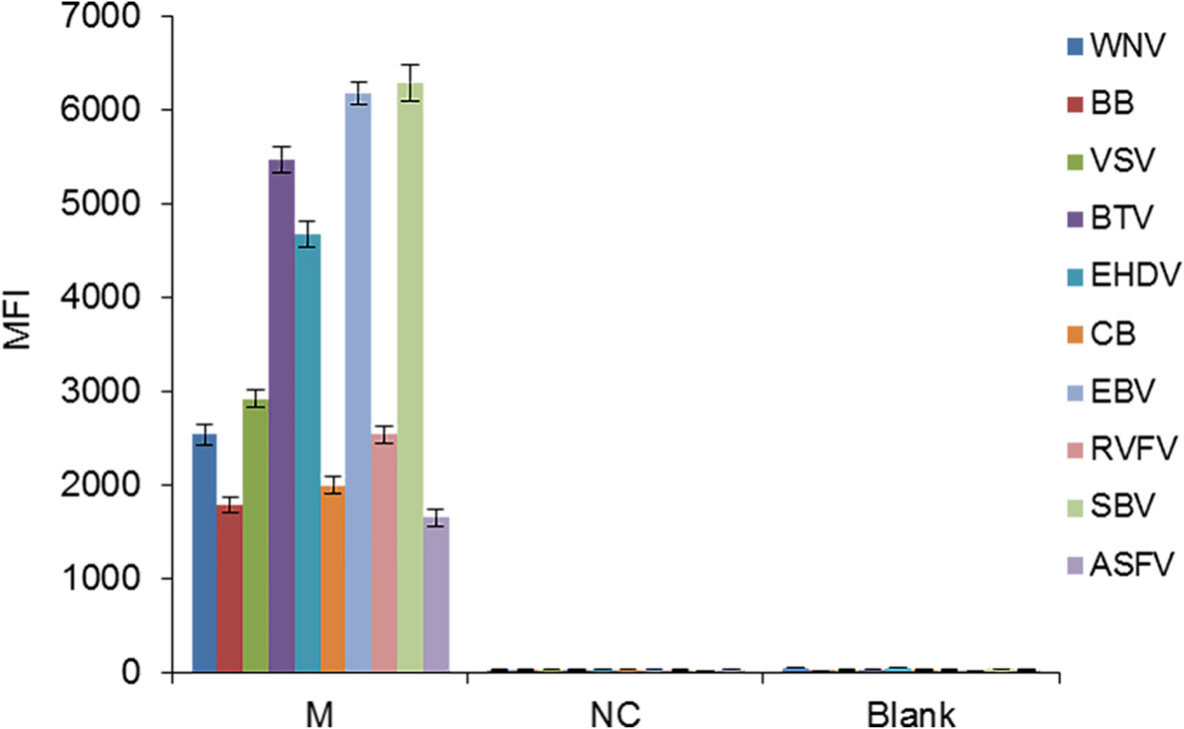
Median fluorescent intensity (MFI) for the 10 insect-borne pathogens. PCR product was replaced with 1 μl of double-distilled H_2_O for the blank control group and 1 μl of negative PCR product for the negative control group. The error bars indicate the standard deviations. *Abbreviations*: M, mixed nucleic acid of the 10 pathogens; NC, negative control; Blank, blank control

To confirm the effectiveness of the optimized liquid array detection system, equal proportion of nucleic acid from the 10 pathogens were mixed and then analyzed using the detection system. The LQRR value was greater than 3, indicating a positive result for the mixed viral nucleic acid (Fig. 3).

**Fig. 4:**
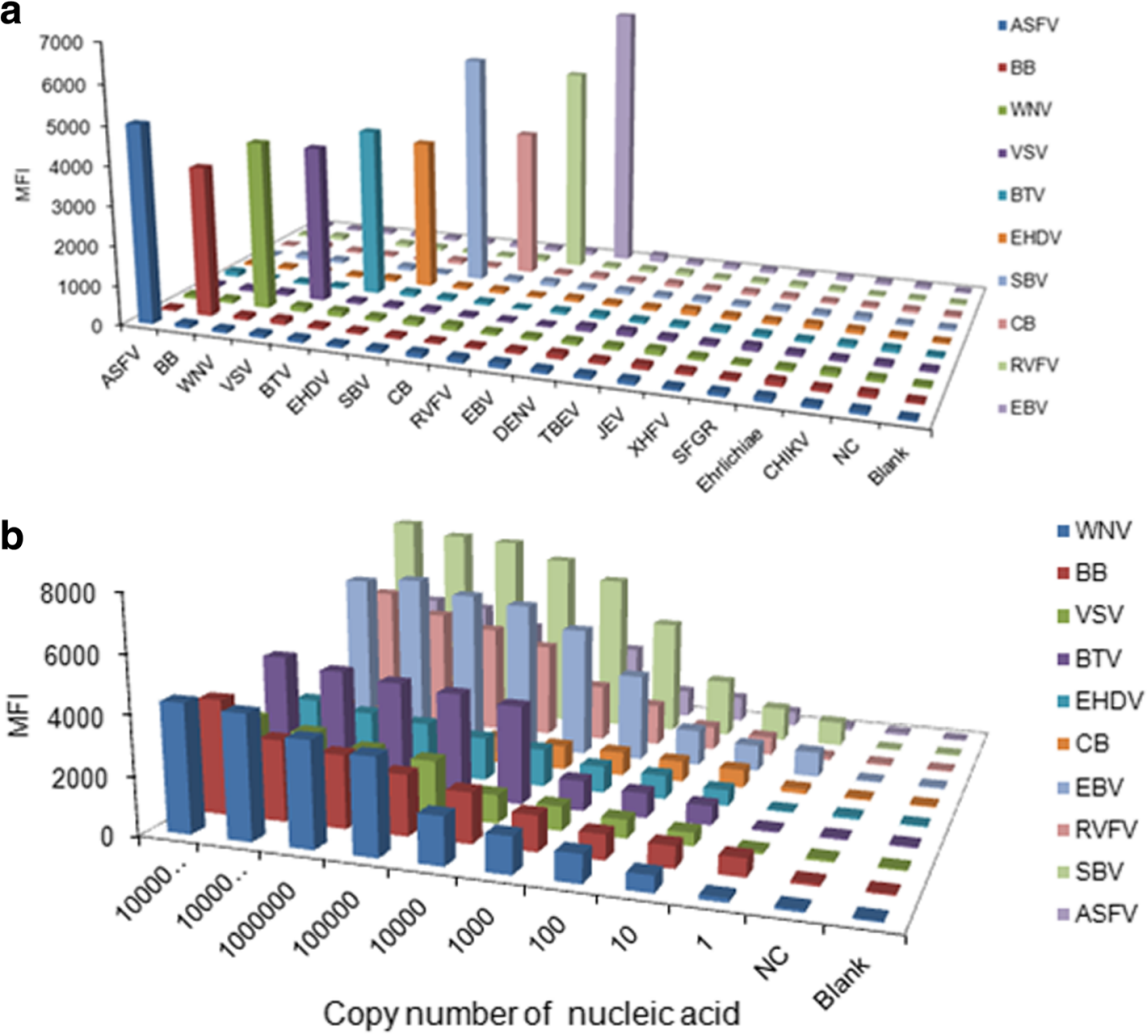
Specificity (**a**) and sensitivity (**b**) of the liquid array detection system. PCR product in the blank control group was replaced with 1 μl of double-distilled H_2_O in the blank control group, and 1 μl negative PCR product in the negative control group. Insect-borne pathogens including dengue virus (DENV), tick-borne encephalitis virus (TBEV), Japanese encephalitis virus (JEV), Xinjiang hemorrhagic fever virus (XHFV), spotted fever group rickettsiae (SFGR), and ehrlichiae and chikungunya virus (CHIKV) were used as controls. *Abbreviations*: NC, negative control; Blank, blank control

PCR products of each type of pathogen were only detected to be positive when hybridized to specific probes coupled with beads, no positive hybridization signals were detected for non-specific probes (Fig. 4a). There was not any cross-hybridization with other insect-borne pathogens including DENV, TBEV, JEV, XHFV, SFGR and CHIKV. This result suggested that the established liquid array detection system had high specificity.

Furthermore, in the liquid array detection system, the LQRR for all of the tested viruses was more than 3 when the copy number for nucleic acid was 10. Strikingly, the LQRR for BB, EBV and SBV was more than 3 when the copy number was 10^1^ (Fig. 4b). By contrast with PCR the pathogens (WNV, BB, ASFV, VSV and BTV) were detected when the copy number for nucleic acid was at least 10^5^, and 10^2^–10^3^ for CB, RVFV, EBV, EHDV and SBV. Collectively, the results suggested that the sensitivity of the liquid array detection system was higher than that observed for the PCR method.

The reproducibility of the detection system was also tested and triplicate experiments confirmed positive results for each of the viruses. Furthermore, the CV was less than 10% (Table 4), indicating that the liquid array detection system has good reproducibility.

**Table 4:** Results of triplicate experiments to test the reproducibility of the liquid array detection system

Insect-borne virus	MFI			SD	CV (%)
	Duplication 1	Duplication 2	Duplication 3		
WNV	4198.00	4054.00	4126.00	72.00	1.75
BB	4293.00	4593.50	4302.00	170.95	3.89
ASFV	1879.00	2019.50	1952.00	70.27	3.60
VSV	2974.50	3153.50	3012.00	94.40	3.09
BTV	8792.00	8811.00	8800.00	9.54	0.10
EHDV	3871.00	3671.50	3796.00	100.77	2.67
CB	1995.00	1950.50	1960.30	23.38	1.19
EBV	7598.50	8062.00	7693.00	244.92	3.15
RVFV	3800.50	4426.00	3912.00	333.64	8.24
SBV	8142.00	8307.00	8423.00	141.21	1.70

*Abbreviations*: *ASFV* African swine fever virus, *BTV* bluetongue virus, *BB Borrelia burgdorferi*, *EHDV* epizootic hemorrhagic disease virus, *VSV* vesicular stomatitis virus, *WNV* West Nile virus, *CB Coxiella burnetii*, *RVFV* Rift Valley fever virus; *EBV* Ebola virus, *SBV* Schmallenberg virus, *MFI* median fluorescent intensity, *SD* standard deviation, *CV* coefficient of variation

### Detection results of clinical samples

Among 3000 clinical samples, 16 tick samples from Inner Mongolia Autonomous Region, Xinjiang Uighur Autonomous Region and Heilongjiang Province were detected to be positive for BB using the optimized liquid array detection system, showed as 2.81% (16/570) infection rate, which was consistent with the results of the detection kit. Furthermore, 53 midge samples from Yunnan Province and Guangdong Province were detected to be positive for BTV, showed as 12.62% (53/420) infection rate, which was also consistent with the results of the detection kit. In addition, 17 tick samples from Inner Mongolia Autonomous Region and Xinjiang Uighur Autonomous Region were positive for CB, showed as 3.62% (17/470) infection rate. Co-infection with BB and CB was found in 5 ticks. All of these samples were reanalyzed using the corresponding gold-standard assays or commercial nucleic acid detection kits, and all of the results were consistent with the findings of the liquid array detection system (Table 3).

## Discussion

The detection of insect-borne diseases is crucial for a rapid response to control such diseases. A high-throughput liquid array panel represents a technological advance that has been widely employed for the detection of various pathogens [16]. However, this methodology has generally been applied for the detection of only a few pathogens. In the present study, we established an optimized multiplexed liquid array detection system that was able to simultaneously detect ten types of insect-borne pathogens. This detection system was confirmed to be specific, sensitive and reproducible.

Some viruses in this study are highly contagious and normally cause human infection. So, the novel virus-like particle or plasmids containing target segments were used as controls to assess the performance of the sample treatment and amplification assay steps.

To ensure the reliability of this detection system, the parameters within the system were optimized. Asymmetric PCR is commonly utilized to produce single-stranded DNA (ssDNA), which can be used for further analysis, such as the detection of hybridization [17]. The ratio of forward/reverse primers is the most critical aspect that influences asymmetric PCR efficiency, and the yield of ssDNA is determined by the concentration of the restrictive primer [18]. In this study, the MFI generated by asymmetric PCR was much higher than that obtained by symmetric PCR. The reason for this may be that the high level of ssDNA products that were extended by the higher concentration of biotinylated reverse primer in turn increased binding efficiency to the complementary probe-labeled microsphere. So the ratio of forward/reverse primers in the asymmetric PCR was optimized, and 1:2 was determined as the optimal ratio.

Furthermore, the conditions of the hybridization, such as hybridization temperature and duration, and the quantity of PCR product, are critical items that ensure the adequacy of a contact reaction [19, 20]. In this study, we optimized the conditions of the hybridization, including the quantity of the PCR product quantity, and the temperature and duration of hybridization. According to the MFI, 2 μl was considered the optimal quantity PCR product, 50 °C was the optimal hybridization temperature and 30 min was the optimal hybridization duration.

High specificity, sensitivity and reproducibility are the desired characteristics of any detection system [21] Strikingly, analysis of the specificity of the detection system failed to detect any non-specific reactions among the 10 types of pathogens tested, indicating that the liquid array detection system had high specificity. This high specificity likely reflects the specificity of the primers and probes used in the PCR and hybridization. Moreover, this detection system displayed high sensitivity with 10 being the lowest copy number detected, which correlated to higher sensitivity than the PCR method. The established detection system was also found to display high reproducibility, which ensured the reliability and stability of this detection method.

This liquid bead array method was used to analyze 3000 clinical samples. The assay detected Lyme disease (LB, caused by BB) and Q fever (QF, caused by CB) among the samples from the Inner Mongolia Autonomous Region and the Xinjiang Uighur Autonomous Region. These two provinces are recognized as the main epidemic areas of LB and QF in China. The results of analysis of these clinical samples indicted that the liquid beads array has promising potential applications in the monitoring of important infectious diseases. Interestingly, it was found the co-infection of *Coxiella burnetii* and *Borrelia burgdorferi* among *Dermacentor nuttalli* and *Ixodes persulcatus* in the Inner Mongolia Autonomous Region. The result indicated that the system could also detect the mixture of different targets.

Notably, the liquid array detection system was able to simultaneously detect 10 different types of pathogens. This number of species of tick-borne pathogens is higher than that detected by any other established method, such as the xMAP liquid array reported to detect nine tick⁃borne pathogens [12], the microwell membrane array for the detection of six species of insect-borne pathogens [11], and the PCR-mass assay for the detection of six insect-borne pathogens [22]. The results of our study suggest that the liquid array detection method might have wider applications.

## Conclusions

This study established a multiplexed PCR-coupled liquid array to simultaneously detect 10 types of insect-borne pathogens. The optimized ratio of forward/reverse primers (1:2) and the conditions of hybridization, including the quantity of PCR product (2 μl), temperature (50 °C) and duration (30 min), were optimized. This improved detection method may be applied for the rapid and effective detection of multiple insect-borne diseases at border ports in China.

## Additional files

Additional file 1: **Figure S1.** Outline of the suspension array method used for the simultaneous and rapid detection of 10 insect-borne pathogens. The hybridization signal of biotinylated PCR product and microsphere beads labeled with specific probes for 10 insect-borne pathogens was detected by instrument. (TIF 220 kb)

Additional file 2: **Figure S2.** Coupling efficiency results for the 10 viral probes and corresponding breads. The error bars indicate the standard deviations. *Abbreviations*: RC-probes, reverse-complement probes; MFI, median fluorescent intensity. (TIF 1380 kb)

## Abbreviations

ASFV: African swine fever virus
BB: *Borrelia burgdorferi*
BTV: Bluetongue virus
CB: Q-fever pathogen *Coxiella burnetii*
CV: Coefficient of variation
EBV: Ebola virus
EHD: Epizootic hemorrhagic disease of deer
EHDV: Epizootic hemorrhagic disease virus of deer
LQRR: Luminex qualitative ratio result
MFI: Median fluorescent intensity
RC-probes: Reverse-complement probes
RVF: Rift Valley fever
RVFV: Rift Valley fever virus
SAPE: Streptavidin-R-phycoerythrin
SB: Schmallenberg disease
SBV: Schmallenberg virus
SD: Standard deviation
ssDNA: Single-stranded DNA
Tm: Melting temperature
TMAC: Tetramethylammonium chloride
VLPs: Virus-like particles
VS: vesicular stomatitis
VSV: Vesicular stomatitis virus
WN: West Nile fever
WNV: West Nile virus
