# Supplementary figures and images for: Establishment and optimization of a liquid bead array for the simultaneous detection of ten insect-borne pathogens
Source: Parasit Vectors. 2018 Jul 31;11:442. doi: 10.1186/s13071-018-2996-0 (PMC6069843; doi:10.1186/s13071-018-2996-0)

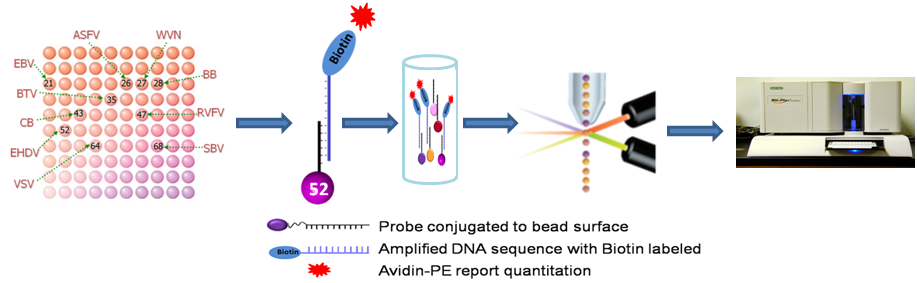

Supplement: Supplementary file 1 — Figure S1. Outline of the suspension array method used for the simultaneous and rapid detection of 10 insect-borne pathogens. The hybridization signal of biotinylated PCR product and microsphere beads labeled with specific probes for 10 insect-borne pathogens was detected by instrument. (TIF 220 kb) [file 13071_2018_2996_MOESM1_ESM.tif]

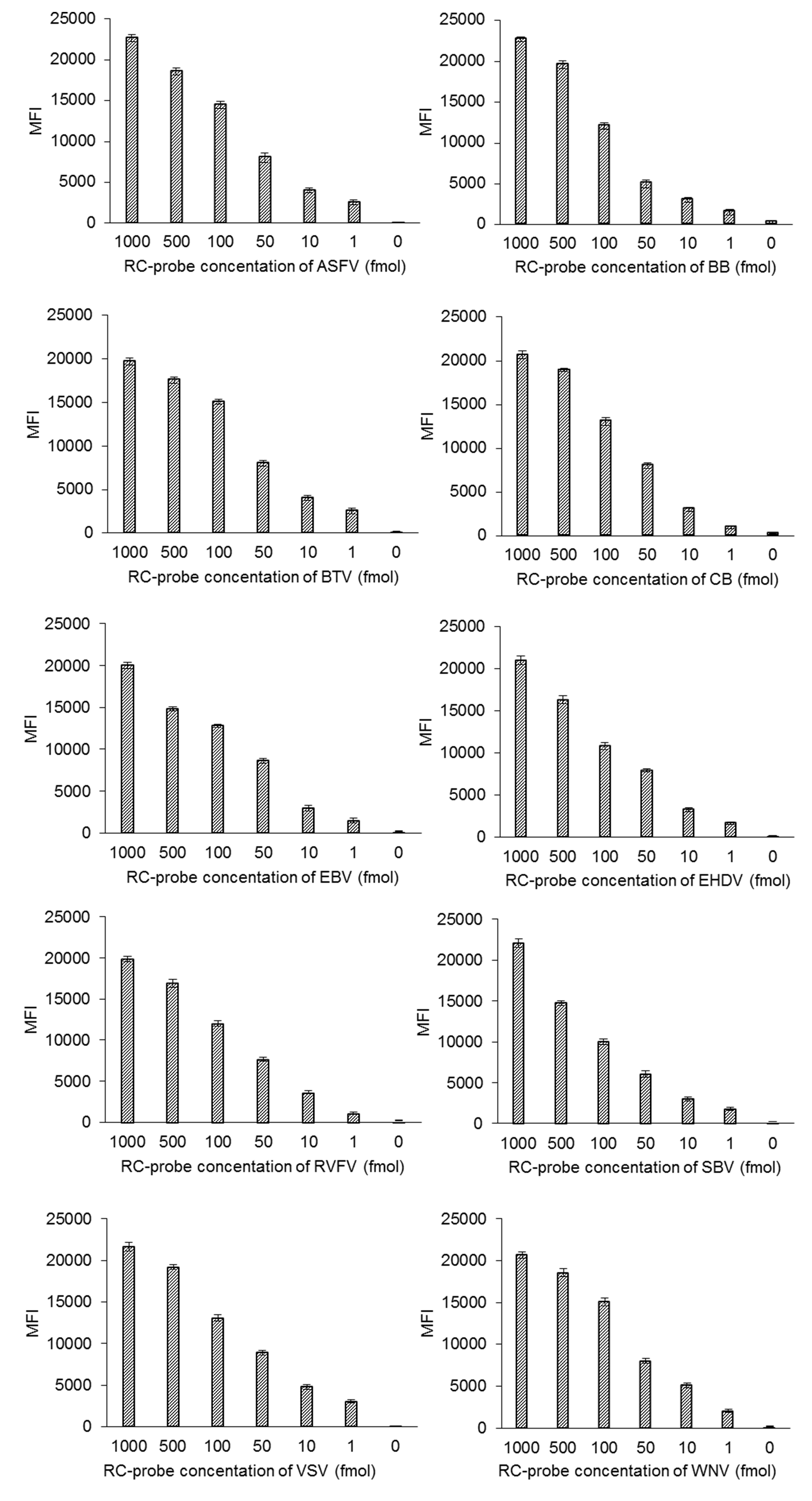

Supplement: Supplementary file 2 — Figure S2. Coupling efficiency results for the 10 viral probes and corresponding breads. The error bars indicate the standard deviations. Abbreviations: RC-probes, reverse-complement probes; MFI, median fluorescent intensity. (TIF 1380 kb) [file 13071_2018_2996_MOESM2_ESM.tif]
